# Domestic Summary

**Published:** 1838-08-15

**Authors:** 


### DOMESTIC SUMMARY.

*Malformation of the Internal Genital Organs in an Adult Female. By* J. B. S. JACKSON, M. D., *of Boston.*

For the following history of the case I am indebted to Dr. Ezra Palmer, Physician to the House of Industry in this city. The patient, a German girl, ætat. 25, entered the House of Industry, on the 13th of last February, under severe salivation; had been taking mercury for a "bilious attack." March 15th she was discharged, but was readmitted on the 1st of April, with œdema of the face and limbs, headach, &c. Understanding from her that she had not menstrurated for five months, Dr. P. considered it a common case of amenorrhœa, and treated her accordingly. Features coarse, but aspect more decidedly feminine than masculine; complexion dark; had always a very timid, abashed, stupid look, and was extremely slow in answering questions; this last seemed to be owing to something more than an ignorance of our language; voice a coarse feminine. Died suddenly on the 16th of April. Since her death Dr. Palmer has succeeded, after much difficulty, in finding a German woman, at whose house the subject of this case staid for some time during the spring. This woman appears to be fully entitled to credit, and gives the following important particulars, which she received from the patient herself. Sailed from Bremen in April, 1837, and arrived in this country on the 3d of September. Before embarking, her health was perfectly good, and she menstruated regularly; was shipwrecked on the passage, besides suffering much from sea-sickness, and after the fright occasioned by this disaster, the catamenia stopped. On her arrival she had œdema of the limbs, spitting of blood, headach, &c., which symptoms were very naturally attributed to the amenorrhœa. An Irishwoman, who laid in an adjoining bed at the House of Industry, being questioned since the death of the patient, stated to Dr. P. that she had heard her speak of having had a child in her own country; as will appear by the dissection, this must have been impossible; and it has been well ascertained, moreover, that she was never married, and that she was the daughter of a respectable farmer; the story, therefore, may have been fabricated by the person who told it, from that love for falsehood which many of the low Irish seem to have.

*April 20th.*—Examination of the body at the dissecting room of the Medical College.

Rather a large frame, and fleshy; lower extremities have somewhat of a masculine appearance, and the hands decidedly so; breasts well developed, as appeared externally and on dissection.

*External organs of generation* perfectly well formed as in any adult female; but, on separating the labia, the vagina, or rather the vulva, is found to be about half an inch in extent, terminating then in a cul de sac, the inner surface having, however, the usual appearance.

The pelvic organs being removed, the rectum was carefully dissected from the bladder, and the vagina was then found to be completely wanting. In the place of it the cellular membrane, to the extent of seven or eight lines laterally, was a little thicker than it was on each side, but without any trace of a cavity.

In the place of the *uterus* are two distinct cornu of a regular, elongated, cylindrical form, going off transversely across the pelvis, and intimately connected with the fundus of the bladder. Something which may be the rudiment of a body of the organ, is sent off by these to be lost insensibly in the cellular membrane which represents the vagina; it is about one and a half inches in extent, slightly thickened, fleshy to the feel, but without any cavity.

*Left cornu* three inches long; three lines in diameter at first, but increases to five lines at the free extremity, which is rather blunt. It consists of a coarse, loose, fibrous structure, of a reddish colour, and having somewhat of a muscular appearance; no trace of cavity. Fallopian tube three and three quarter inches long, and of about the usual size; fimbriated extremity large and free. Ovary about one-third as large again as usual, of a flattened form, smooth on the surface, and very flaccid to the feel, as if it contained many vesicles; though it was not cut open, it hangs free in the cavity of the pelvis, in a sort of broad ligament, but much less to the left side than usual; ligament connecting it with the cornu quite distinct.

*Right cornu* one and a half inches in length, or more than twice that of the other, for the most part about three lines in diameter, but increasing to six at its ovarian extremity. It differs from the other cornu in structure, being white, rather dense, and more resembling the common "uterine tissue." No trace of cavity; after it leaves the fundus of the bladder, it penetrates through the muscles, and appears at the external inguinal ring; there it gives off a fallopian tube three inches in length, of about the usual size, having a free and well developed fimbriated extremity. The ovary, which lays fairly in the groin between Poupart's ligament and the superficialis fascia, is one and one-third of an inch long, two-thirds of an inch wide, and one-third of an inch thick, of a very regular oval form, a little flattened, rather firm, very white, and much more resembling a testicle than an ovary; it was nearly surrounded by a serous membrane, forming a sort of tunica vaginalis, though between the opposing surfaces there were numerous adhesions; the fallopian tube was mostly, if not entirely, bound down by adhesions, besides being very much contorted, so that the limits between it and the cornu were not clearly made out till after some dissection.

The parts are now in the cabinet of the Society for Medical Improvement.

The kidnies were much and very peculiarly diseased, but did not exactly resemble any of the forms described by Dr. Bright. The bladder, which was large and collapsed, contained three or four ounces of urine, some of which being tested by heat coagulated strongly.

The other organs had been removed.

*Am. Jour. Med. Sciences, Aug.* 1838.

